# Abortion of Typhoid Fever

**Published:** 1895-10

**Authors:** 


### Abortion of Typhoid Fever.

Every now and then there comes to the front some writer with a plan to abort typhoid fever; it is of more than annual occurrence. We can remember,—long before the days of microbes and antiseptics,--the late venerable Professor Fenner—the founder of the *New Orleans Medical Journal*, had a theory, and a practice too, by which he claimed that typhoid fever could be aborted. He used to say, as a clincher to his argument, "all the other doctors have cases of typhoid fever; I have none; why? because I cut it short in the beginning." Well—they didn't laugh at him, exactly; it was, they thought, a harmless hobby, and they had too much respect for him to laugh at him, but nobody believed him. Very recently an old gentleman read a paper on the subject at the American Medical Association meeting, at Hot Springs, giving *his* plan. Notwithstanding his age and venerable looks, he was laughed at. But, it is no laughing matter. Investigators are on the right track,—the subject has elicited serious attention, and many begin to believe that sooner or later a plan will be devised, a rational treatment—a treatment based upon exact knowledge of the pathology—whereby the course of the disease will be much shortened and its character changed. Several interesting papers on the subject have appeared recently; one, which strikes us as coming very near the secret of the matter, was read by Dr. John Aulde, of Philadelphia, before the Mississippi Valley Medical Association, at its 21st annual meeting at Detroit, early in September (ult.), and of which a lengthy abstract appeared in the *New York Medical Journal* of September 14. It will be seen that a new remedy is introduced; it plays an important role, in accordance with bacteriological developments; a feature not heretofore taken into consideration in the treatment, —*nuclein*. From this abstract we reproduce the following. The writer said:

As a preliminary to these remarks it would be appropriate to mention some of the objections to the routine methods which had been so long advocated, although it was not deemed advisable to enter into a study of its pathology, because the pathological conditions in typhoid fever were too well known at the present day. The routine treatment of this disease was devoted principally to the disinfection of the intestinal tract, the profession being under the impression that the micro-organisms found there a suitable soil for their rapid multiplication. This assumption, however, was true only in part. In the first part, while it was true that the micro-organism associated with this disease found a suitable nidus for its reproduction in Peyer's patches, we must bear in mind that inflammation of Peyer's patches was not always attended by ulceration; hence the morbid process was not actually in the intestinal tract, as usually understood. So long as the micro-organisms were confined to these bodies, although the disease was of intestinal origin, the effects were constitutional

—due to the absorption of poisonous products, not only from the affected areas, but from the intestinal tract as well. It was principally for this reason that intestinal antiseptics had failed; and, for the same reason, it should be added, we might expect benefit from the employment of remedies which assisted in rendering the contents of the small intestine aseptic, since from the foregoing explanation we could understand how advantageous they would prove. Salol, beta-napthol, and guaiacol, were efficient remedies of this class, and it had lately been suggested that the latter may be used externally with good results.

The first-mentioned of these remedies, salol, was effective in the treatment of typhoid fever because when it entered the intestine it was broken up into its constituent elements, salicylic acid and phenol, and both ingredients were eliminated principally by the kidneys. But the benefits were often more apparent than real, and there came a time when these remedies acted unfavorably, owing to to their poisonous influence upon the renal structures, and they had to be abandoned. Moreover, it was now well known that carbolic acid was a most objectionable cardiac depressant, and the faithful clinician found that, however beneficial its effects, salol could not be given continuously in the treatment of this disease. Beta-naphthol was not open to this objection, but its employment was often contraindicated by reason of the pain and burn which followed its administration, and, in addition to this, patients rebelled against it, owing to its taste. This latter objection might be obviated by administering the drug in capsules, but even then we did not overcome the objection first noted. Nevertheless, it was, he says, the ideal remedy for this disease, provided we assumed it to be a disorder confined to the intestine, but it was not strictly an intestinal affection, as had previously been stated.

We had in copper arsenite a remedy which fulfilled every requirement, and, moreover, it had been pretty thoroughly tested clinically. Since the autumn of 1888, when the author had first brought its virtues to the attention of the profession, he had used it constantly with the most satisfactory results. In addition to his own experience, he had received numbers of flattering reports from other physicians in general practice, notably one from Dr. A. H. Thomas, of Hurley, Wis., who had passed through an epidemic of typhoid fever which occurred in Hurley and Ishpeming, Mich., during the summer of 1893. Dr. Thomas had reported (*American Therapist*, December, 1893) ninety cases treated, in which copper arsenite had constituted the principal medicament, with but a single death, and that from intestinal hemorrhage. Since the autumn of 1888, the author had never failed to abort or shorten the course of typhoid fever by the use of copper arsenite, together with the administration of other indicated remedies presently to be mentioned, and he believed that this disease could be arrested at any stage.

This latter statement was so sweeping in its character that an explanation should be added. In its incipiency, and probably for the first week of an attack, typhoid fever was specific, but after this period it was usually composite in character; in other words, it was a mixed infection, due to the effect of the disease upon the functions of elimination. Now, when the statement was advanced that typhoid fever could be arrested at any stage, it meant that the typhoid or specific nature of the infection could be caused to disappear, when there remained a simple continued fever. Any one having a case of typhoid fever under observation would find it an easy matter to verify this.

The plan of treatment was then briefly outlined as follows: When a case of suspected typhoid fever came under observation the patient is confined to bed, a suitable diet ordered, and a careful record of the morning and evening temperature kept. As a rule, copper arsenite in doses of one one-hundredth of a grain was given at intervals of from four to six hours while the patient was awake. Should there be evidences of hepatic complications, mercury biniodide was substituted for the copper salt, one one-hundredth of a grain being administered at intervals of two or three hours for one or two days, and that was generally sufficient to correct or remove this complication for the time being. It might be necessary to repeat this medication, but the mercurial should not be permitted to supplant the copper salt. When the patient was restless or sleepless, it might be expedient to administer small doses of the bromides, or codeine sulphate might be substituted, one-fifth of a grain at intervals of two hours during the afternoon. In addition to this, the author had found nuclein solution, the animal product, most effective in restoring the functional activity of the glandular system, from one-third to one minim, at intervals of from two to four hours. In serious cases, or when the patient had advanced to the second week of the disease, both remedies should be given hypodermically, preferably in the following manner: A tablet of chemically pure copper arsenite containing one grain of the salt was dissolved in four ounces of boiled water, and to this mixture dilute hydrochloric acid was added drop by drop until the mixture becomes clear, being thoroughly agitated meantime. Each thirty minims of this clear solution carried appoximately a milligramme (one sixty-fifth of a grain), and this amount could be injected under the skin at some indifferent point night and morning. The nuclein solution was given in doses of from two to five minims (five to ten drops), diluted with a sufficient quantity of sterilized water to make a syringeful, and introduced subcutaneously in the same manner twice a day.

The simplicity of the treatment was all that could be desired, and its efficiency would be apparent to those who had the temerity to test its virtues. Several of the author's professional friends had reported remarkable results from the use of nuclein solution alone, although in most instances it had been tried only in the hopeless cases, after the apparent failure of the approved methods.

Anodynes were used solely as a temporary expedient for the purpose of quieting the irritant effect of the poisons upon the nervous system; the mercurial was employed for its influence upon the hepatic function, which was apt to become deranged, owing to the extra work thrown upon the liver in eliminating or destroying the poisons; the copper arsenite acted as an intestinal antiseptic, through its influence upon the nervous system and upon the protoplasm at the point of elimination—namely, the epithelial cells of the intestinal mucous membrane. Through its irritant effect upon protoplasmic cells throughout the body, being administered in extremely small doses, it acted continuously as a stimulant, augmenting cellular activity in every part. The nuclein complemented this action by enacting the role of a ferment; but, in addition to this, it established an artificial leucocytosis, an important function which had been demonstrated to be absent in typhoid fever. This latter was a feature which had been overlooked in the treatment of typhoid fever. For the lack of proper nourishment, phagocytotic activity was held in abeyance in this disease; the multinuclear white blood-corpuscles, being unprovided with suitable pabulum, were unable to produce the needed defensive proteids, of which nuclein was the chief, and as a consequence metabolism was hindered, and waste products accumulated, so that to the specific infection were added the disorders resulting from suboxidation and defective elimination.

By this plan of treatment typhoid fever could be arrested, if taken in the early stages, within a few days, or at most in less than a week. When the treatment was adopted during the second week of the disease or subsequently, the peculiar character of the affection was changed; the temperature fell, the patient experienced a feeling of well-being, threatened complications subsided, and recovery took place, relapses being unknown.

Again, on the subject of aborting typhoid fever, W. B. Thistle, M. D., LL. D., London, of the University of Toronto, has had three papers; the third one, "Third Report on Eliminative and Antiseptic Treatment of Typhoid Fever," appears in the *New York Medical Record* of September 14th. He believes that the disease can be aborted by a combination of antiseptic and eliminative treatment. From his article, we reproducethe following reasoning and conclusions. We believe it will be read with much interest and benefit by our readers:

In my first paper, I stated that I believed all the indications furnished by a study of the morbid process were met by the adoption of a plan of treatment embracing three distinct heads or principles: Elimination, antisepsis, and dilution. I still adhere to that belief. Elimination is accomplished by free and continuous purgation, as well as by the flushing action of large quantities of water on kidneys and bowels. By the use of purgative medicines, the infective process is disturbed in several ways: 1. Bacilli are carried out of the intestine together with the toxic substances produced by them. 2. Poison held in solution by the body fluids escapes with the free secretion into the intestine: and besides, at frequent intervals a quantity of what, in this instance, must be extremely toxic bile, is swept away instead of continuing in the circuit from the liver to the intestine and back again. If constantly relieved in this way, the liver can more frequently perform its *role** of standing guard and intercepting toxic substances which would otherwise reach the general circulation. 3. Infection of intestinal glands is limited, or, in other words, the source of supply from which bacilli and poison are carried from the intestine is cut off. That is, just as you can and do limit the dose of poison received by the system generally, so do you limit the local dosage and consequent injury of the intestinal glands by the same means. This surely requires no argument, it is evident, if only one stops to recall the manner in which infection takes place. In the one case, lessened dose means milder symptoms, and in the other the smaller the local dose the less severe the local lesion.

Concerning toxin already absorbed and in contact with the tissues, held in solution by the body fluids, some portion of it must be removed with the secretion poured into the intestine, when stimulated with purgative medicines, as well as that drained off through the kidneys. There is nothing unusual in such a claim. We have many examples of removal of poisonous material from the body by similar procedure.

By repeating this process it certainly tends to prevent a dangerous accumulation of toxin in the system, and at the same time prevents, or tends to prevent, local lesion in the intestine from becoming sufficiently great to destroy a vessel, or extend entirely through the intestinal wall. It must not be forgotten that tissue resistance is increased just as the tissues are freed from the effect of the poison. Hence, the tissue cells are in this way rendered more capable of resisting and destroying the bacilli already present in them. It is obvious, too, in order to escape harm, the earlier elimination is brought about and the more constantly it is secured, the better, whether with reference to general symptoms or local lesion. In many instances, I believe, brisk purgation in the early stage carries out so much of the culture, and relieves the tissue to so great an extent that the remaining bacilli are destroyed by the liberated tissue, and the fever is aborted.

When this is not accomplished, or when the case is not seen

*Bouchard: Auto-intoxication in Disease.

early, then purgation must be prompt and energetic enough to relieve dangerous conditions, and continuous enough to keep the dose of poison below a harmful point, if possible, until such time as immunity is reached. In judging of the amount of poison present, attention should not be exclusively directed to one or two symptoms, but the entire list of symptoms should be considered.

Coming now to the second feature in treatment, that is, attempting to destroy micro-organisms by antiseptics, there can scarcely be doubt about the possibility of doing this to some degree. It is quite possible to completely deodorize the contents of the intestine by means of salol or salicylate of bismuth, and, no doubt, by other agents, as any one can demonstrate. But to be efficient, antiseptics should be used in association with purgatives for the following reasons:

1. Just as it is easier to approximately sterilize an abscess-cavity after first having emptied it, so is it to derive benefit from intestinal antiseptics if the intestine be first cleared of its contents.

2. Intestinal antiseptics, while lessening the production of poison by destroying bacilli, yet could have no possible effect on the poison already in the intestine, but in many instances might themselves add to the toxins present.

3. Much larger quantities of antiseptics can be used without poisonous symptoms arising, if, at the same time, elimination by the bowels is continuously maintained. Any benefit derived from an antiseptic is obtained at once, and if it is speedily cleared away much, which would otherwise be absorbed, escapes with the contents of the bowels.

The remaining factor in treatment is clearly indicated, for the local effect of the poison depends upon its degree of concentration, as well as upon duration of contact.

Injection of large quantities of fluid is a necessary adjunct to elimination. Fluid drained off, continuously carrying with it the poisonous material it contains, must be replaced. If this were not done, the tissues would suffer from lack of fluid, and the toxin remaining would assume a more concentrated, and therefore a more active, form. It might be well, at this point, to consider elimination by purgation in its relation to certain accidents of the disease, *i. e.*, hemorrhage and perforation. I do not propose to enter into the question at length, but shall content myself by pointing out that, if it is true that the local lesion is proportionate to the local dosage and duration of contact, and if it is true that the toxin and bacilli, which would otherwise reach the lymphatic glands, can be carried out by purgation, then it must be that elimination by purgation tends to lessen the occurrence of both hemorrhage and perforation.

In the late cases the indications are the same. If a vessel is already necrosed, hemorrhage is unavoidable; if not already necrosed, the way to prevent it becoming so is by removing the poison. With reference to this point I beg to refer to the paper in the *Canadian Practitioner* of April, 1893, or to the *Medical Record* of March 10, 1894, where I have discussed this question at length. I still maintain the same conclusion, that purgation at no time causes perforation or homorrhage, but at all times tends to prevent its occurrence. Misconception regarding this matter was the great barrier which interposed whenever an attempt was made to follow in treatment the indications furnished by study of the morbid process. So great was the dread of these two accidents that the fact that the great majority of fatalities were due, not to hemorrhage and perforation, but to toxæmia, was lost sight of. A study of the cases I have to report with reference to this point, will, I believe, convince the most sceptical that formerly a misconception did exist.

So much for meeting the indications furnished by considerations of what has been determined concerning micro organisms on paper or in theory. How does it work out in actual practice? Precisely as it does in theory.

I have to report 172 cases with a mortality of five, or three per cent.

